# Subclinical congestion assessed by whole-body bioelectrical impedance analysis in HFrEF outpatients

**DOI:** 10.1007/s12471-025-01962-3

**Published:** 2025-06-23

**Authors:** Bruno Bragança, Mauro Moreira, Rafaela G. Lopes, Inês G. Campos, José Luís Ferraro, Ricardo Barbosa, Sónia Apolinário, Licínia Aguiar, Magda Soares, Patrícia Silva, João Azevedo, Aurora Andrade

**Affiliations:** 1 Department of Cardiology, Unidade Local de Saúde Tâmega e Sousa (ULSTS), Penafiel, Portugal; 2 Laboratório de Farmacologia e Neurobiologia, Instituto de Ciências Biomédicas Abel Salazar, Universidade do Porto (ICBAS-UP), Porto, Portugal; 3 RISE-Health, Porto, Portugal

**Keywords:** HFrEF, Heart failure, Electric impedance, BIA, residual congestion, Subclinical congestion

## Abstract

**Background:**

Persistent congestion in heart failure (HF) carries a dismal prognosis. Bioimpedance analysis (BIA) non-invasively identifies extracellular water (ECW) redistribution associated with acute HF. However, its role in detecting subclinical congestion in HF outpatients still needs to be explored.

**Methods:**

Eighty-three adult outpatients with HFrEF were recruited for a single-center prospective study. Segmental multi-frequency BIA was used to assess body composition and the extracellular-to-total body water ratio (ECW/TBW), a marker of fluid redistribution. Subclinical congestion was defined as ECW/TBW_z‑score_ > 2 without clinical signs of congestion. The primary outcome was a composite of all-cause death and worsening HF (WHF) events.

**Results:**

In this cohort, 57% of patients had subclinical congestion. Higher congestion grades were associated with age, female sex, and comorbidities. ECW/TBW_z‑score_ correlated linearly with NT-proBNP levels and low muscular indexes were associated with congestion severity. During a median follow-up of 10 months, 27% of patients experienced the primary outcome, mostly WHF events. Both subclinical and clinical congestion were independently associated with an increased risk of the primary outcome, with hazard ratios (HR) of 9.4 (1.04–85.1; *p* = 0.046) and 17 (1.11–261; *p* = 0.042), respectively. NT-proBNP and ECW/TBW_z‑score_ showed similar power in predicting the outcome.

**Conclusions:**

BIA detects subclinical congestion—a condition highly prevalent in outpatients with HFrEF. An increased ECW/TBW ratio correlates with established markers of congestion and is associated with adverse events in this population. These findings support the integration of BIA into routine HF care; however, further studies are needed to establish the clinical benefits of BIA-guided management and its impact on patient outcomes.

**Supplementary Information:**

The online version of this article (10.1007/s12471-025-01962-3) contains supplementary material, which is available to authorized users.

## What’s new

- Whole-body BIA detects subclinical congestion in outpatients with HFrEF.
- Subclinical congestion is highly prevalent, affecting 6 out of 10 HFrEF outpatients.
- Subclinical congestion is associated with worsening heart failure (WHF) events and a worse prognosis.

## Introduction

**Fig. 1:**
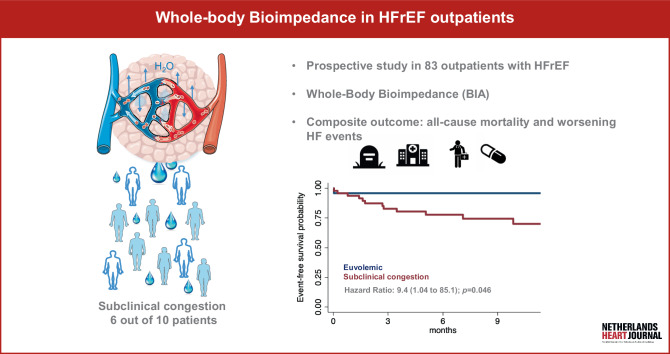
Subclinical congestion and prognosis by whole-body bioimpedance in HFrEF outpatients

Heart failure (HF) is characterised by recurrent episodes of decompensation and frequent hospital admissions. Congestion is the hallmark of HF decompensation and results from hemodynamic changes and deleterious activation of neuro-hormonal systems that promote the expansion and redistribution of extracellular water (ECW) [1]. An increase in intraventricular filling pressures and redistribution of ECW mark the onset of a ‘congestion cascade’ [2]. In daily clinical practice, the identification of congestion typically relies on physicians’ clinical skills. Decongestive therapy is a critical step in transitioning care from hospital to community settings for patients with acute HF (AHF); however, subtle forms of congestion are often missed [3, 4]. Several diagnostic tools have been implemented to improve fluid status assessment, including natriuretic peptides, cardiothoracic imaging, and hemodynamic monitoring via implantable devices [1, 5]. However, these methods are not always easily accessible, can be costly, and some carry inherent risks. Despite advances, subclinical congestion remains problematic and carries a dismal prognosis [3, 6]. Bioimpedance analysis (BIA) is a non-invasive, rapid, and reproducible method for assessing body composition and fluid status. BIA measures the resistance and reactance of a low-level alternating current passing through a network of body resistors (fluids) and capacitors (cell membranes) at specific frequencies. Multifrequency BIA allows characterisation of water content in the extracellular (low-frequency) and intracellular compartments (high-frequency), as well as other body components such as body fat mass (BFM) and fat-free mass (FFM) [7]. While there is solid evidence supporting the use of BIA in AHF, its role in chronic HF (CHF) remains underexplored [8]. Therefore, we evaluated the use of BIA in CHF outpatients with reduced left ventricular ejection fraction (HFrEF), aiming to identify subclinical congestion and evaluate its prognostic impact (Fig. 1).

## Methods

### Study design and population

From February to May 2023, we consecutively recruited HFrEF patients, regularly followed at the HF outpatient clinic from the Unidade Local de Saúde Tâmega e Sousa, for BIA. Patients were prospectively followed up until July 2024 or until the occurrence of the primary outcome. The primary outcome was a composite of all-cause mortality and worsening HF events (WHF: hospitalisation, unplanned visits, or diuretic uptitration). Eligible adults (≥ 18 years) with a prior diagnosis of HFrEF and LVEF < 50% at the time of recruitment were included, whereas unstable patients, those requiring inotropic support or dialysis, those who were pregnant or had limb amputations, or those unable to provide informed consent were excluded. Patients with cardiac implantable electronic devices were not excluded, as no significant safety issues have been reported with BIA [9]. The study protocol was previously reviewed and approved by the local Ethics Committee (protocol number: 13/2023) and conducted in accordance with the Declaration of Helsinki.

### Whole-body bioimpedance analysis

Body composition was assessed using a segmental multi-frequency (1 kHz to 3 MHz) whole-body BIA device (InBody BWA 2.0, Seoul, Korea). Briefly, four connected clamp electrodes connected to an octa-polar 16-point electrode were placed on the patients’ ankles and wrists (Fig. S1). BIA was conducted in the morning after patients had rested for five minutes. All patients were weighed on calibrated scales, wearing light clothing and no shoes. Low appendicular skeletal muscle index (ASMI) was defined according to the EWGSOP guidelines [10]. Absolute ECW volume overload (ECW_overload_) was calculated as previously described [11]. All researchers had significant experience in the daily care of HF patients and had received specific training in BIA.

### Clinical assessment

Patients’ electronic medical records were reviewed at the end of the follow-up period to collect baseline data and identify the occurrence of events of interest. Biochemical analyses were conducted at the hospital laboratory using standard methods; only biochemical data obtained within two weeks of BIA assessment were included. Baseline echocardiographic LVEF was included if it was within 6 months of BIA assessment.

### Congestion status

Cardiologists and nurses examined all patients on the day of BIA as part of their routine HF care. Cardiologists involved in the clinical assessment of the patients were blinded to the BIA data. Patients were categorised according to their volemic status into three groups: euvolemic, subclinical congestion, and clinical congestion. Clinical congestion was defined by the presence of at least one of the following clinical signs: jugular venous distension, pulmonary rales, and lower limb oedema. BIA-derived congestion was defined by an ECW/TBW two standard deviations (SD) above the mean (z-score > 2) from a healthy population database provided by the device vendor. Subclinical congestion was ECW/TBW_z‑score_ _>_ _2_ and no clinical signs of congestion at physical examination.

### Statistical analysis

Continuous data are presented as mean ± SD, and the relative frequencies of discrete variables as percentages (%). Comparisons between groups were performed using one-way ANOVA, Kruskal-Wallis, Student’s *t*-test, or Pearson’s chi-squared test, as appropriate. Hazard ratios (HRs) and confidence intervals (CIs) for outcomes were calculated using Cox regression models and compared via the log-rank test. Multivariate analyses were performed to adjust HRs for clinically relevant baseline variables. A receiver operating characteristic (ROC) analysis was used to compare the predictive accuracy of the ECW/TBW_z‑score_ with NT-proBNP. A *p*-value < 0.05 was considered significant. Statistical analyses were conducted using Stata software, version 16.1 (StataCorp® LLC, College Station, TX, USA).

## Results

### Study population

A total of 83 Caucasian patients were recruited for this study. Baseline characteristics are presented in Tab. 1. Approximately one-quarter of the patients had simultaneously four cardiovascular risk factors (CVRFs). With regard to class I disease-modifying drugs, 86% of the patients were receiving quadruple therapy for HFrEF. BIA revealed that 52% of body weight was water; with 61% of this water being located intracellularly (ICW). A low ASMI was present in 14% of patients (Tab. S1).

**Table 1:** Clinical characteristics of the HF population by volemic status at baseline

	*Total*	*Euvolemic*	*Subclinical congestion*	*Clinical congestion*	* p value*
Number of patients, *n* (%)	83 (100)	24 (28.9)	47 (56.6)	12 (14.5)	
Age, years (sd)	64.8 (11.0)	58.9 (8.1)	66.3 (10.7)	72.3 (10.7)	< 0.001
Male sex, *n* (%)	60 (72.0)	20 (83.0)	36 (77.0)	4 (33.0)	0.004
*Cardiovascular risk factors*					
Overweight/Obesity, *n* (%)	49 (59.0)	12 (50.0)	32 (68.0)	5 (42.0)	0.14
Hypertension, *n* (%)	54 (65.0)	10 (42.0)	35 (74.0)	9 (75.0)	0.017
Diabetes, *n* (%)	36 (43.0)	6 (25.0)	22 (47.0)	8 (67.0)	0.046
Dyslipidemia, *n* (%)	68 (82.0)	19 (79.0)	35 (74.0)	10 (83.0)	0.92
Smoke (active), *n* (%)	14 (17.0)	7 (29.0)	6 (13.0)	1 (8.0)	0.31
Cerebrovascular disease, *n* (%)	16 (19.0)	3 (12.0)	9 (19.0)	4 (33.0)	0.33
Coronary artery disease, *n* (%)	41 (49.0)	11 (46.0)	25 (53.0)	5 (42.0)	0.71
Chronic kidney disease, *n* (%)	50 (61.0)	7 (29.0)	32 (70.0)	11 (92.0)	< 0.001
Atrial Fibrillation, *n* (%)	30 (36.0)	6 (25.0)	16 (34.0)	8 (67.0)	0.044
Ischaemic HF, *n* (%)	38 (46.0)	10 (42.0)	24 (51.0)	4 (33.0)	0.49
Systolic blood pressure, mm Hg (sd)	114 (18)	117 (14)	111 (19)	117 (19)	0.31
Diastolic blood pressure, mm Hg (sd)	65 (12)	68 (13)	64 (12)	63 (12)	0.36
Heart rate, bpm (sd)	70 (13)	69 (12)	70 (14)	70 (6)	0.98
*NYHA class, n (%)*					
I	9 (11.0)	5 (21.0)	4 (9.0)	0 (0)	0.12
II	65 (78.0)	18 (75.0)	40 (85.0)	7 (58.0)	0.12
III	9 (11.0)	1 (4.0)	3 (6.0)	5 (42.0)	< 0.001
LVEF, % (sd)	33.4 (10)	33.5 (12)	34.4 (9.9)	29.5 (7.9)	0.36
Complete left bundle block (LBBB), *n* (%)	21 (26.0)	8 (35.0)	9 (19.0)	4 (33.0)	0.30
Complete right bundle block (RBBB), *n* (%)	10 (12.0)	3 (13.0)	6 (13.0)	1 (8.0)	0.91
*Blood analysis*					
Nt-proBNP, pg/mL (sd)	2236 (3875)	501 (453)	2592 (4380)	4339 (4366)	0.014
Creatinine, mg/dL (sd)	1.28 (0.48)	1.06 (0.22)	1.34 (0.47)	1.46 (0.74)	0.022
Sodium, mM (sd)	139 (2.4)	139 (2.6)	139 (2.3)	138 (2.6)	0.35
Haemoglobin, g/dL (sd)	14.3 (1.8)	15.3 (1.1)	14.0 (1.6)	13.4 (2.4)	0.002
Albumin, g/dL (sd)	4.2 (0.4)	4.4 (0.3)	4.2 (0.5)	4.0 (0.4)	0.22
Gamma-glutamyl transferase, IU/L (sd)	75.8 (84.6)	69.8 (66.6)	61.9 (53.5)	135.8 (159.5)	0.033
Alkaline Phosphatase, IU/L (sd)	70.5 (43.4)	67.2 (49.1)	68.9 (31.0)	82.4 (66.2)	0.61
*HF therapy, n (%)*					
Loop diuretics	51 (61.0)	8 (33.0)	32 (68.0)	11 (92)	0.001
ACEi/ARB	25 (30.0)	6 (25.0)	13 (28.0)	6 (50.0)	0.26
ARNi	54 (65.0)	17 (71.0)	32 (68.0)	5 (42.0)	0.18
Beta-blocker	74 (89.0)	23 (96.0)	41 (87.0)	10 (83.0)	0.43
MRA	72 (87.0)	22 (92.0)	40 (85.0)	10 (83.0)	0.69
SGLT2i	83 (100)	24 (100)	47 (100)	12 (100)	–
Pacemaker	3 (4.0)	0 (0)	1 (2.0)	2 (17.0)	0.029
ICD	14 (18.0)	5 (21.0)	8 (17.0)	2 (17.0)	0.92
CRT‑D	11 (13.0)	3 (12.0)	6 (13.0)	2 (17.0)	0.93

*ACEi* Angiotensin converting enzyme inhibitors, *ARB* Angiotensin receptor blockers, *ARNi* Angiotensin receptor/neprilysin inhibitor, CRT‑D Cardiac Resynchronisation Therapy Device with ICD, *LVEF* Left Ventricular Ejection Fraction, *HF* Heart Failure, *ICD* Implantable cardioverter-defibrillator, *MRA* Mineralocorticoid receptor antagonist, *NT-proBNP* N‑terminal pro b‑type natriuretic peptide, *SGLT2i* Sodium-glucose cotransporter-2 inhibitors

*Notes*: Ischemic HF when compromise of LVEF was attributed to bstructive CAD. Chronic kidney disease (CKD) was defined as an estimated glomerular filtration rate (GFR) < 60 mL/min/1.73 m^2^

### Congestion status

Subclinical congestion was present in 57% of patients (Tab. 1). The concordance between BIA and physical examination was 42%; one patient was classified as clinically congestive despite normal BIA values (Fig. S2). The degree of congestion was associated with age, female sex, New York Heart Association (NYHA) functional class, and several comorbidities such as diabetes, hypertension, CKD, and atrial fibrillation (AF). Congestion was also associated with a decrease in muscle mass indexes (Tab. S1). ECW/TBW indexes correlated with NT-proBNP (Tab. S1 and Fig. S3). There were no statistical differences in ECW/TBWz-score (*p* = 0.585), ECW_overload_ (*p* = 0.774), and NT-proBNP (*p* = 0.222) between congestive groups.

### Subclinical congestion and prognosis

**Fig. 2:**
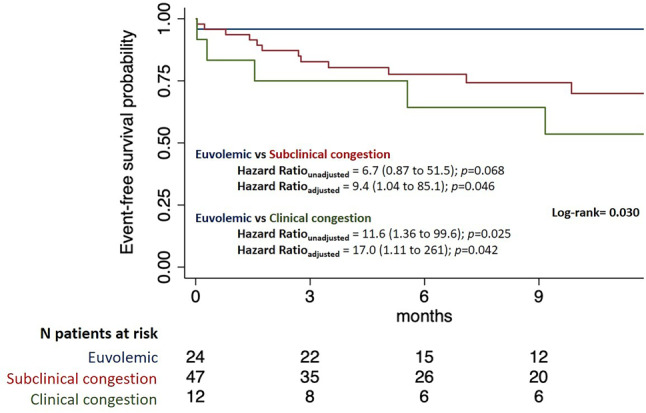
Event-free survival analysis

**Fig. 3:**
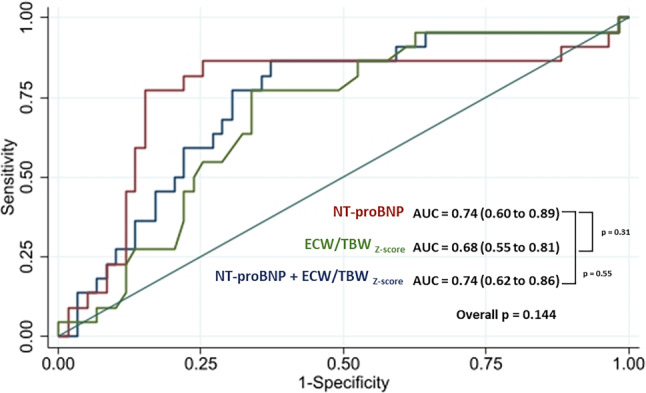
Prediction of the composite outcome by NT-proBNP and ECW/TBW_z‑score_

During a median follow-up of 10 months, the composite outcome occurred in 22 patients, primarily due to WHF events (Tab. 2). The time to HF worsening was approximately 5 months in both congestive groups. Congestion was associated with higher event rates. CKD, NYHA functional class, haemoglobin, and the ECW/TBW_z‑score_ were associated with the outcome (Tab. S2). These variables, along with other clinically relevant variables including age, sex, diabetes, hypertension, AF, HF aetiology, LVEF, and NT-proBNP, were included in a multivariate analysis. In the event-free survival analysis, subclinical and clinical congestion were independently associated with a higher incidence of the primary outcome compared to euvolemic patients, with an HR of 9 and 17, respectively (Fig. 2). The probability of the outcome increased linearly with both the ECW/TBW_z‑score_ and NT-proBNP (Fig. S4). There was no significant difference in the predictive accuracy between NT-proBNP and ECW/TBW_z‑score_, and their combination did not improve composite outcome prediction (Fig. 3).

**Table 2:** Outcome events during follow-up

	*Total*	*Euvolemic*	*Subclinical congestion*	*Clinical congestion*	* p value*
Follow-up, days (sd)	237 (144)	269 (141)	225 (140)	220 (164)	0.440
Composite outcome, *n* (%)	22 (27.0)	1 (4.0)	15 (32.0)	6 (50.0)	0.006
All-cause death, *n* (%)	5 (6.0)	0 (0)	2 (4.0)	3 (25.0)	0.009
Cardiovascular death, *n* (%)	3 (4.0)	0 (0)	2 (4.0)	1 (8.0)	0.420
HF hospitalisation, *n* (%)	9 (11.0)	0 (0)	7 (15.0)	2 (17.0)	0.130
Unplanned visit, *n* (%)	3 (4.0)	0 (0)	2 (4.0)	1 (8.0)	0.420
Uptitration of diuretics, *n* (%)	16 (19.0)	1 (4.0)	10 (21.0)	5 (42.0)	0.023
Worsening HF events, *n* (%)	20 (24)	1 (4.0)	15 (32.0)	6 (50.0)	0.006
Time to first event, days (sd)	151 (158)	1 (‑)	163 (166)	145 (152)	0.610

## Discussion

### BIA in the assessment of subclinical congestion

BIA-estimated hypervolemia correlates with signs of congestion in AHF, including raised levels of natriuretic peptides, and peripheral or lung oedema [12]. While BIA may not add significant value when congestion is clinically evident, it may enhance its detection in subtler forms. In this cohort, subclinical congestion was more prevalent (57%) than previously reported (17%) [13]. This low-grade congestion may not be detected on physical examination, even by experienced physicians [4], highlighting the importance of multiparametric congestion assessment using biomarkers and non-invasive tools [5, 13–15]. BIA appears more accurate in detecting peripheral oedema than natriuretic peptides [16]. In this study, although NT-proBNP correlated with ECW/TBW, it did not differ between congestive groups, suggesting that mild-to-moderate elevation of natriuretic peptides may not effectively distinguish these states [17], a situation not rarely seen in clinical practice. Although half of the cohort was overweight/obese, which limits volemic assessment by physical examination, this did not correlate with ECW/TBW. BIA has shown good performance in detecting congestion and guiding diuretic therapy in obese HF patients [18], thereby broadening its potential in the highly heterogeneous HFpEF population. Diagnosing HFpEF in patients with chronic unexplained dyspnoea remains challenging, as it often relies on probability estimates derived from a combination of multiple variables. Even an elevated NT-proBNP is insufficient for HFpEF diagnosis, particularly in obese patients [19]. We speculate that BIA could aid in the reclassification of patients with an intermediate HFpEF probability by identifying subtle congestion, as demonstrated in a recent computed tomography algorithm for detecting pulmonary congestion [20]. This approach could potentially reduce the need for the often inaccessible invasive hemodynamic stress testing and facilitate HFpEF diagnosis.

### Subclinical congestion and prognosis

BIA-guided therapy enables a safer transition of AHF patients to outpatient care by detecting residual congestion [15, 21]. BIA-estimated congestion predicts WHF events in outpatient settings [13, 22–24]. Notably, LVEF was not associated with the degree of congestion or outcomes [17]. A recent prospective study in HFrEF outpatients showed that BIA-estimated hypervolemia was independently associated with WHF events, outperforming other surrogate markers of congestion, including NT-proBNP, and indexes of LV filling pressure and central venous pressure (CVP). In contrast, in our study, the predictive accuracy of NT-proBNP and ECW/TBW_z‑score_ was comparable. Although Rodriguez-Lopez’s study did not specifically address subclinical congestion, a volume excess of 1.2 L predicted WHF events [13]. Similarly, in our study ECW/TBW_z‑score_ _>_ _2_ corresponds to an average absolute volume overload of 1 L. In addition, all patients identified with congestion had ECW/TBW > 0.39, a cutoff previously linked to adverse HF outcomes [23]. Challenging this evidence, Curbelo et al. found that NTproBNP, lung ultrasound and CVP, but not ECW/TBW, were associated with WHF events in CHF outpatients [25], though their study reported 60% ECW, which is a paradoxical water distribution, indicating a potential methodological issue.

### BIA and patterns of congestion

HF presents with two primary congestion patterns—intravascular and interstitial—which often coexist [1]. In HFrEF, interstitial congestion predominates and results from saturation of different fluid buffering mechanisms, such as venous capacitance, lymphatic drainage, and interstitial hydrostatic pressure [26]. Conversely, rapid fluid redistribution into the intravascular space, with increased pulmonary and cardiac filling pressures, usually presents with acute pulmonary oedema [1]. Identifying the dominant congestion phenotype has therapeutic implications, particularly for diuretic and vasodilator therapy. While BIA correlates with body weight and lung ultrasound changes, Curtain et al. showed that it does not correlate with single measurements of pulmonary capillary wedge pressure (PCWP) [27]. This discrepancy can be attributed to the use of BIA devices that provide only arbitrary units of impedance, which are not suitable for single-point measurements. However, this limitation can be overcome by tracking relative impedance changes over time or by quantifying fluid volumes in specific compartments using advanced BIA devices, as demonstrated in our study.

### BIA and body composition in heart failure

HF progression is associated with a loss of muscle mass and a ‘counterintuitive’ reduction in both ECW and TBW can be observed [28]. As muscle mass is a major contributor to TBW, ECW/TBW indexes should be the preferred metric to assess fluid redistribution and congestion in HF. Although muscle function was not assessed in this study—a key requirement for diagnosing sarcopenia [10], 14% of the patients had low ASMI, consistent with previous reports in CHF cohorts [29]. Furthermore, low ASMI correlated with the degree of congestion and other surrogate markers of HF severity. However, it is important to note that current BIA devices may underestimate the true prevalence of sarcopenia in HF, particularly in congestive states where BIA-derived muscle mass tends to be overestimated [30]. BIA allows the identification of patients at risk of sarcopenia who may benefit from interventions, such as dietary and exercise programs [28]. Another valuable BIA parameter is phase angle (PhA), a marker of cellular health, with PhA < 4.2° linked to poor nutritional status and adverse events in HF [14]. In our study, PhA was inversely correlated with congestion severity. However, larger studies are needed to validate PhA as as a reliable indicator of cellular health in HF patients, particularly in cohorts with comparable fluid status.

## Limitations

The recruitment of patients from a single specialised HF clinic may limit the generalizability of our findings; however, this study reflects real-world data from HFrEF patients under standard-of-care. The small cohort size may have increased the risk of type II errors, though ECW/TBW_z‑score_ remained independently associated with outcomes after adjusting for confounders. Despite the prospective design of the study, some clinical variables were not collected at the time of BIA, which may have introduced bias; however, this was mitigated by including data close to recruitment. While BIA is validated for assessing volume status, subclinical congestion detected by BIA was not compared with other diagnostic methods discussed in this study. Lastly, there is no universally accepted cut-off for defining congestion using BIA. We addressed this issue using state-of-the-art BIA technology and comparing variations relative to a healthy population.

## Conclusion

BIA demonstrates sensitivity in detecting hypervolemia that may be missed during clinical evaluation. Subclinical congestion is prevalent among HFrEF outpatients and is independently associated with adverse outcomes. The integration of BIA into routine outpatient care may improve HF management and patient outcomes, but these need to be confirmed in further studies.

## Supplementary Information

**Fig. S1 **Bioelectrical Impedance Analysis (BIA) measurement procedure

**Fig. S2 **Contingency table comparing volume status assessment by BIA and physical examination.

**Fig. S3 **Relationship between ECW/TBW_Z‑score_ and NT-proBNP

**Fig. S4 **Probability of the composite outcome according to NT-proBNP and ECW/TBW_z‑score_

**Tab. S1 **Whole-body BIA characteristics of the HF population by volemic status at baseline

**Tab. S2 **Predictors of the Composite Outcome
