# Supplementary figures and images for: Subclinical congestion assessed by whole-body bioelectrical impedance analysis in HFrEF outpatients
Source: Neth Heart J. 2025 Jun 23;33(7-8):239–45. doi: 10.1007/s12471-025-01962-3 (PMC12274172; doi:10.1007/s12471-025-01962-3)

**Fig. S1** Bioelectrical Impedance Analysis (BIA) measurement procedure


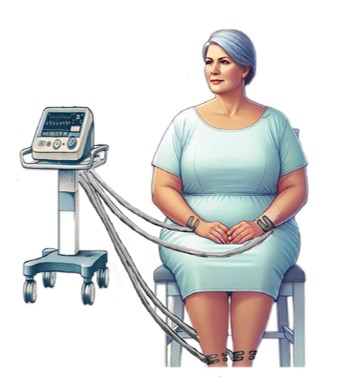

Supplement: Supplementary file 1 — Fig. S1 Bioelectrical Impedance Analysis (BIA) measurement procedure [file 12471_2025_1962_MOESM1_ESM.docx]

**Fig. S2** Contingency table comparing volume status assessment by BIA and physical examination.


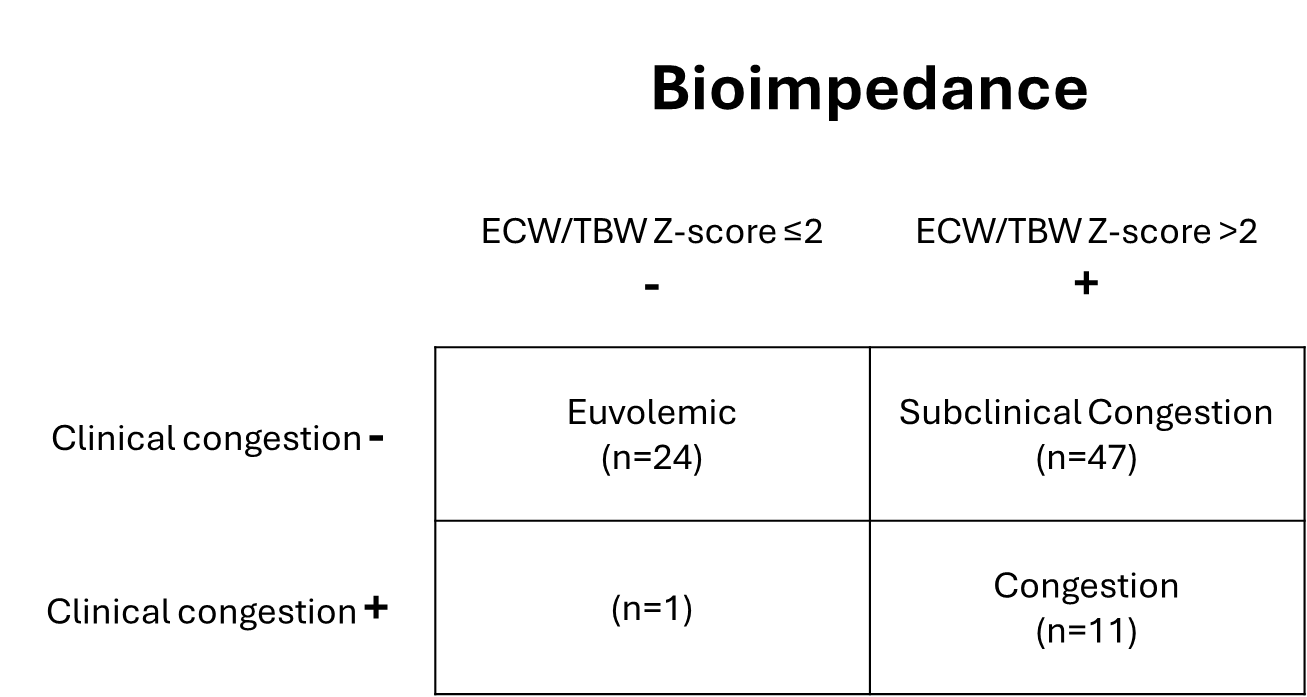

Supplement: Supplementary file 2 — Fig. S2 Contingency table comparing volume status assessment by BIA and physical examination. [file 12471_2025_1962_MOESM2_ESM.docx]

**Fig. S3** Relationship between ECW/TBWZ-score and NT-proBNP


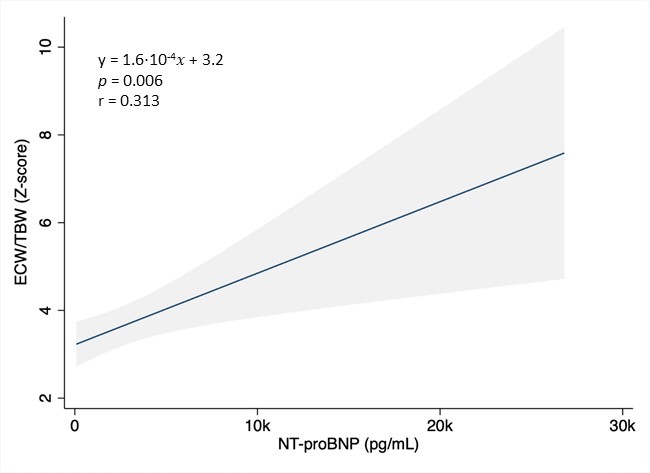

Supplement: Supplementary file 3 — Fig. S3 Relationship between ECW/TBWZ‑score and NT-proBNP [file 12471_2025_1962_MOESM3_ESM.docx]

**Fig. S4** Probability of the composite outcome according to NT-proBNP and ECW/TBWz-score


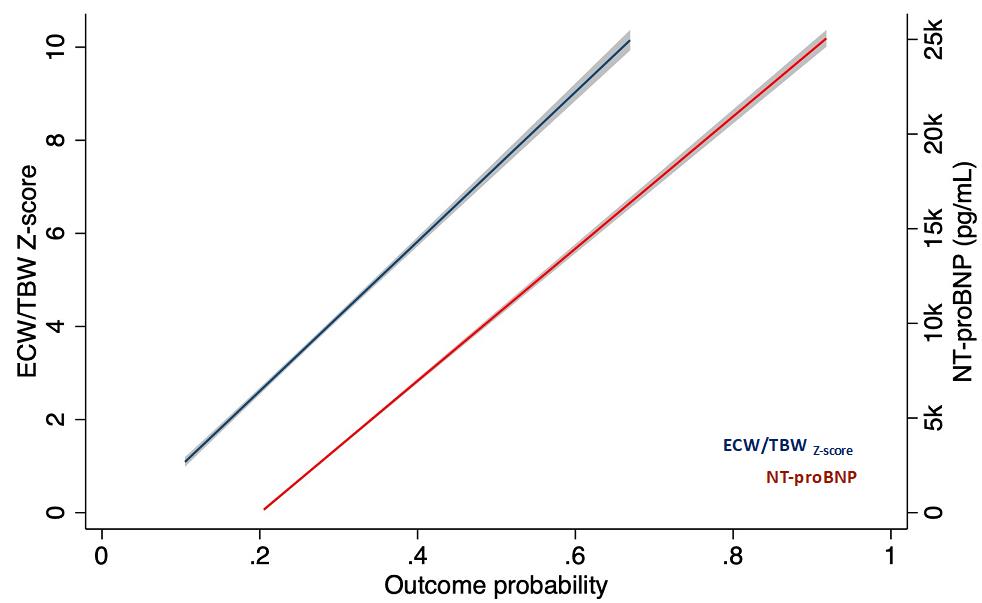

Supplement: Supplementary file 4 — Fig. S4 Probability of the composite outcome according to NT-proBNP and ECW/TBWz‑score [file 12471_2025_1962_MOESM4_ESM.docx]
