# Supplementary material for: Subclinical congestion assessed by whole-body bioelectrical impedance analysis in HFrEF outpatients
Source: Neth Heart J. 2025 Jun 23;33(7-8):239–45. doi: 10.1007/s12471-025-01962-3 (PMC12274172; doi:10.1007/s12471-025-01962-3)
Supplement: Supplementary file 5 — Tab. S1 Whole-body BIA characteristics of the HF population by volemic status at baseline [file 12471_2025_1962_MOESM5_ESM.docx]

**Tab. S1** Whole-body BIA characteristics of the HF population by volemic status at baseline

|  | *Total* | *Euvolemic* | *Subclinical congestion* | *Clinical congestion* | *p value* |
| --- | --- | --- | --- | --- | --- |
| Weight, Kg (sd) | 74.5(12.7) | 78.2 (12.3) | 74.2 (11.1) | 68.1 (17.1) | 0.074 |
| Height (Ht), cm (sd) | 165.3 (7.7) | 167.9 (6.2) | 165.3 (7.8) | 160.3 (7.7) | 0.018 |
| Body mass index, Kg/m2 (sd) | 27.2 (3.8) | 27.7 (3.9) | 27.1 (3.2) | 26.4 (5.8) | 0.59 |
| Intracellular water (ICW), L (sd) | 23.5 (5.3) | 26.1 (5.4) | 23.0 (4.6) | 19.9 (5.3) | 0.002 |
| Extracellular water (ECW), L (sd) | 14.9 (2.8) | 15.3 (2.1) | 15.1 (2.9) | 13.2 (3.5) | 0.080 |
| Total body water (TBW), L (sd) | 38.3 (7.7) | 41.4 (7.0) | 38.1 (7.4) | 33.1 (8.8) | 0.009 |
| TBW/Weight, % (sd) | 51.6 (6.4) | 53.2 (6.6) | 51.4 (6.1) | 49.1 (7.0) | 0.18 |
| ECW/TBW, % (sd) | 39.0 (2.0) | 37.3 (2.6) | 39.6 (1.1) | 40.0 (1.0) | <0.001 |
| ECW/TBW Z-score (sd) | 3.2 (3.3) | 0.32 (4.4) | 4.35 (1.8) | 4.66 (1.5) | <0.001 |
| ECW/TBW >39%, n (%) | 40 (48) | 0 (0) | 31 (66.0) | 9 (75.0) | <0.001 |
| ECW overload, L (sd) | 0.51 (1.60) | -0.66(2.45) | 0.98 (0.68) | 1.04 (0.51) | <0.001 |
| Segmental ECW/TWB, % (sd) |  |  |  |  |  |
| Right arm | 38.3 (0.7) | 37.6 (0.5) | 38.5 (0.6) | 38.6 (0.4) | <0.001 |
| Left arm | 38.2 (0.7) | 37.6 (0.7) | 38.4 (0.6) | 38.5 (0.6) | <0.001 |
| Trunk | 39.1 (2.2) | 37.2 (2.9) | 39.8 (1.2) | 40.3 (0.9) | <0.001 |
| Left leg | 39.2 (2.1) | 37.3 (2.7) | 39.9 (1.2) | 40.2 (0.8) | <0.001 |
| Right leg | 39.0 (2.4) | 36.9 (3.3) | 39.7 (1.2) | 40.3 (0.9) | <0.001 |
| Body fat mass, Kg (sd) | 22.3 (8.2) | 21.7 (9.0) | 22.4 (7.3) | 23.0 (10.7) | 0.89 |
| Body fat mass/weight, % (sd) | 29.8 (8.9) | 27.3 (9.1) | 30.2 (8.4) | 33.2 (9.5) | 0.16 |
| Soft lean mass, Kg (sd) | 49.1 (10.2) | 53.4 (9.4) | 48.7 (9.5) | 42.2 (11.3) | 0.007 |
| Fat-free mass, Kg (sd) | 52.2 (10.8) | 56.5 (9.9) | 51.8 (10.1) | 45.0 (11.9) | 0.008 |
| Minerals, Kg (sd) | 3.7 (0.7) | 3.87 (0.7) | 3.71 (0.7) | 3.3 (0.8) | 0.11 |
| Proteins, Kg (sd) | 10.1 (2.3) | 11.3 (2.4) | 10.0 (2.0) | 8.6 (2.3) | 0.003 |
| Skeletal muscle mass, Kg (sd) | 28.6 (6.8) | 32.0 (7.1) | 28.1 (6.0) | 23.9 (7.0) | 0.002 |
| Appendicular skeletal muscle index (ASMI), Kg/m2 (sd) | 7.46 (1.2) | 7.9 (1.0) | 7.4 (1.1) | 6.5 (1.6) | 0.002 |
| Low ASMI, n (%) | 12 (14) | 0 (0) | 9 (19) | 3 (25) | 0.051 |
| Resistance (R/Ht), Ω/m (sd) | 228 (48) | 218 (29) | 222 (39) | 270 (83) | 0.004 |
| Reactance (Xc/Ht), Ω /m (sd) | 22.9 (6.3) | 28.0 (7.4) | 20.7 (4.2) | 21.6 (5.0) | <0.001 |
| Phase angle (PhA), ◦ (sd) | 5.85 (1.6) | 7.34 (1.8) | 5.38 (1.1) | 4.7 (0.85) | <0.001 |
