# Supplementary material for: Subclinical congestion assessed by whole-body bioelectrical impedance analysis in HFrEF outpatients
Source: Neth Heart J. 2025 Jun 23;33(7-8):239–45. doi: 10.1007/s12471-025-01962-3 (PMC12274172; doi:10.1007/s12471-025-01962-3)
Supplement: Supplementary file 6 — Tab. S2 Predictors of the Composite Outcome [file 12471_2025_1962_MOESM6_ESM.docx]

**Tab. S2** Predictors of the Composite Outcome

| Variable | *Odd ratio* | *95% CI* | *p value* |
| --- | --- | --- | --- |
| Age | 1.04 | 0.99 – 1.09 | 0.150 |
| Female | 1.31 | 0.45 – 3.89 | 0.616 |
| Hypertension | 1.61 | 0.56 – 4.71 | 0.381 |
| Diabetes | 1.44 | 0.54 – 3.83 | 0.465 |
| Overweight/Obesity | 1.70 | 0.61 – 4.76 | 0.312 |
| Chronic Kidney Disease | 5.44 | 1.45 – 20.4 | 0.012 |
| Atrial fibrillation | 2.21 | 0.82 – 5.99 | 0.119 |
| Non-ischaemic HF | 0.48 | 0.18 – 1.30 | 0.148 |
| NYHA | 7.75 | 1.96 – 30.7 | 0.004 |
| LVEF | 1.00 | 0.94 – 1.94 | 0.861 |
| Gamma-GT | 1.00 | 1.00 – 1.01 | 0.136 |
| Haemogloblin | 0.73 | 0.54 – 0.98 | 0.037 |
| Albumin | 0.77 | 0.17 – 3.43 | 0.735 |
| NT-proBNP (ng/mL) | 1.14 | 0.99 – 1.32 | 0.075 |
| TBW | 0.96 | 0.90 – 1.03 | 0.243 |
| ECW | 0.93 | 0.78 – 1.11 | 0.449 |
| ICW | 0.93 | 0.84 – 1.03 | 0.182 |
| ECW/TBW Z-score | 1.29 | 1.01 – 1.63 | 0.039 |
| EVW/TBW Z-score > 2 | 12.7 | 1.60 – 101 | 0.016 |
| Body fat mass | 1.04 | 0.98 – 1.10 | 0.220 |
| Fat-free mass | 0.97 | 0.93 – 1.02 | 0.241 |
| ASMI | 0.76 | 0.50 – 1.15 | 0.194 |
| Phase angle | 0.73 | 0.49 – 1.08 | 0.111 |
